# Infants exposed to maternal type 1 diabetes: intrauterine epigenetic modifications and neurological development

**DOI:** 10.3389/fendo.2026.1759949

**Published:** 2026-02-10

**Authors:** Nieves Luisa González-González, Enrique González-Dávila, José Ramón Castro-Conde, Candelaria González-Campos, Olivia Orribo-Morales, Carlos Flores, José Miguel Lorenzo-Salazar, Rafaela González-Montelongo, Adrián Muñoz-Barrera, Erika Padrón-Pérez, Nazaret Villalba-Martín, Abraham Acevedo-Arozena, Laura Tascón-Padrón, Marina Armas-González

**Affiliations:** 1 Obstetrics and Gynecology Department, Facultad de Ciencias de la Salud, Sección de Medicina. University of La Laguna (ULL), San Cristóbal de La Laguna, Tenerife, Spain; 2 Mathematics, Statistics and Operations Research Department, IMAULL. University of La Laguna (ULL), San Cristóbal de La Laguna, Tenerife, Spain; 3 Pediatrics Department, Canary Islands University Hospital, La Laguna San Cristóbal de La Laguna, Tenerife, Spain; 4 Obstetrics and Gynecology Department, Canary Islands University Hospital, San Cristóbal de La Laguna, Tenerife, Spain; 5 Research Unit, Hospital Universitario Ntra. Sra. de Candelaria, Instituto de Investigación Sanitaria de Canarias (IISC), Santa Cruz de Tenerife, Spain; 6 CIBER de Enfermedades Respiratorias (CIBERES), Instituto de Salud Carlos III, Madrid, Spain; 7 Genomics Division, Instituto Tecnológico y de Energías Renovables (ITER), Santa Cruz de Tenerife, Spain; 8 Research Unit, Hospital Universitario de Canarias, ITB-ULL and CIBERNED, San Cristóbal de La Laguna, Tenerife, Spain; 9 Department of Obstetrics and Prenatal Medicine, University Hospital Bonn, Bonn, Germany; 10 Department of Child and Adolescent Psychiatry. Hospital General Universitario Gregorio Marañón, Madrid, Spain

**Keywords:** cord blood, DNA, epigenetic, gene, maternal diabetes, methylation, neurodevelopment, type 1 diabetes

## Abstract

**Background:**

The relationship between the neurodevelopment in infants exposed to maternal Type-1 diabetes and changes in fetal DNA methylation has not yet been investigated.

**Aim:**

This hypothesis-generating study, we aimed to determine whether neurodevelopmental outcomes in offspring from mothers with Type-1 diabetes are associated with intrauterine epigenetic changes in fetal DNA.

**Material and Methods:**

We conducted a prospective, pilot case-control study, comparing infants exposed to maternal Type-1-diabetes with control infants. Cord blood DNA samples were analyzed using the TruSeq-Methyl-Capture-EPIC-Kit, covering over 3.3 million CpGs. The Bayley-III Scales were used to assess infant neurodevelopment, and the scores were correlated with the newborn DNA methylation data.

**Results:**

In infants exposed to maternal diabetes, we identified 108 differentially methylated genes enriched in pathways crucial for neurodevelopment: Vocal, Imitative and Observational Learning, Synapse Organization, and Neurogenesis. The greatest methylation differences were observed in differentially methylated regions (DMRs) annotated to key neurological genes: *MYT1L* (21.61%, q=1.97E-07), *NRXN1* (12.30%, q=5.04E-75), *SHANK3* (11.62%, q=9.81E-06) and *KIRREL3* (7.35%, q= 1.53E-23). Both, *NRXN1* and *SHANK3*, were enriched across all identified neurodevelopmental pathways. At two years of age, the infants exposed to maternal Type-1 diabetes scored significantly lower on the Bayley-III Scales across the cognitive, language, and motor domains. Methylation values across loci annotated to ten neurodevelopment-associated genes were linked to Bayley-III cognitive, language, and/or motor domain scores—with *MYT1L* and *NRXN1* showing significant correlation with the Bayley-III language domain score.

**Conclusions:**

While further confirmation is needed, we provide the first results supporting the hypothesis that neurodevelopmental alterations observed in offspring of mothers with Type-1 diabetes are potentially associated with DNA methylation changes during intrauterine life which can be identified at birth.

## Introduction

Carbohydrate intolerance is the medical complication that most often complicates pregnancy. The most common type is gestational diabetes (GDM) (2-32%). Unlike GDM, Type-1-diabetes is present before pregnancy, and is much less frequent (0.2-1%), and much more serious. Moreover, Type-1diabetes is not related to maternal age or obesity, as GDM (1).

Cognitive and psychomotor impairments, as well as lower intelligence quotient scores, have been observed in infants exposed to maternal diabetes, and these effects may persist into adolescence (2). Maternal hyperglycemia is thought disrupt fetal brain development through pathways involving neuroinflammation, oxidative stress, nutrients and hormone delivery, and epigenetic modifications. Optimal glycemic control during pregnancy is associated with reduced incidence and severity of these complications (3).

Epigenetics refers to heritable changes in gene function that occur without altering the underlying DNA sequence. During fetal development, epigenetic processes are crucial. They orchestrate tissue-specific gene expression, cellular differentiation, and overall developmental programming in response to both genetic predispositions and external conditions, including the intrauterine metabolic environment (4).

Accumulating evidence suggests that GDM triggers epigenetic changes in fetal DNA associated with an increased risk for metabolic and cardiovascular diseases, and neurologic disorders, in childhood and adult life. These epigenetic modifications have been identified in multiple tissues, including placenta, cord blood, and amniocytes (5).

To the best of our knowledge, the relationship between the neurodevelopment of infants exposed to maternal Type-1 diabetes (IMT1-diabetes) and changes in fetal DNA methylation has not yet been investigated. The only study conducted has exclusively assessed changes related to cardiovascular and metabolic diseases in offspring (6). Epigenetic marks could potentially serve as biomarkers for early intervention strategies in newborns at high risk for neurological disorders.

Our aim was to determine if the neurodevelopmental outcomes of offspring from mothers with Type-1 diabetes are associated with intrauterine epigenetic changes in fetal DNA. Given that, no previous studies have explored this specific association, we designed this pilot study as a hypothesis-generating analysis.

## Materials and methods

This is a prospective, hypothesis-generating pilot case-control study that included 10 IMT1-diabetes and 10 infants of healthy mothers (IHM) recruited at the University Hospital of Canary Islands, Spain, between June 2021 and January 2024. Pregnant women were matched 1:1 using a nearest neighbor based on pre-pregnancy BMI, stratified according to the World Health Organization (WHO) categories, and race/ethnicity. This case-control design was fundamental for establishing a physiological baseline to isolate maternal T1D-specific effects from standard population variations.

Inclusion criteria were: Singleton gestation, absence of maternal pathology, mode of delivery (vaginal or elective cesarean section under regional anesthesia), absence of medical or obstetrics complications and of toxic habits (tobacco, drug abuse, other medication than insulin), and gestational-age >37 weeks. Exclusion criteria: Birthweight of IHM <10^th^ or >90^th^ percentile, according to customized birthweight curves (7), congenital malformations, chromosomal abnormalities and admission to neonatal unit for special care. Normal glucose tolerance of healthy mothers was ensured according to the NDDG criteria (8). HbA1c-levels were determined in mothers with type1-diabetes throughout the pregnancy.

This study was conducted in accordance with the Declaration of Helsinki and was approved by the Clinical Ethics Committee of the Canary Islands University Hospital Complex, Spain (CHUC-201817). All mothers signed a written consent forms.

### Whole-genome bisulfite sequencing (WGBS) analysis

Cord blood was collected immediately after birth in 3 ml K3-EDTA tubes in the delivery room and immediately stored at −80°C until DNA extraction. DNA of cord blood samples was purified using Blood genomicPrep Mini Spin Kit (Cytiva Amersham™). DNA concentrations were measured on the Qubit 3.0 fluorometer using the Qubit dsDNA HS Assay (Thermo Fisher Scientific).

### Bisulfite conversion and Methyl-Seq procedures

Bisulfite conversion of DNA and library preparation were done using the TruSeq-Methyl-Capture-EPIC Library Prep Kit (Illumina Inc.) (9), which targets 3.34 million CpGs sites, following the manufacturer’s recommendations. Library sizes were assessed with a 4200 TapeStation (Agilent Technologies) and the concentration determined with the Qubit dsDNA HS Assay (Thermo Fisher Scientific). Sequencing was conducted at the Instituto Tecnológico y de Energías Removables (ITER, Santa Cruz de Tenerife. Canary Islands, Spain) with a NovaSeq 6000 Sequencing System (Illumina Inc.) using 110 bp paired-end single-indexed reads along with 1% of a PhiX Control-V3 (Illumina Inc.) (9).

### Bioinformatics analysis to obtain the cytosine methylation

We used bcl2fastq v2.20 (10) to perform sample demultiplexing and Bismark v0.24.0 (11) for bisulfite mapping and methylation calling against the GRCh37/hg19 reference genome. Bismark-based pipeline transformed the sequence and reads into fully bisulfite-converted forward (C->T) and reverse read (G->A) conversion of the forward strand versions. Then, transformed reads were aligned to similarly converted versions of the reference genome (also C->T and G->A converted). The best alignment from the four alignment processes against the bisulfite genomes were compared to the normal genomic sequence and the methylation state of all cytosine positions in the read was inferred.

CpGs sites were considered differentially methylated (DM) if differences were > 15% and the q-value < 0.01. Promoters, regions and islands were considered DM if differences were ≥ 1% and q-value < 0.01. DM CpGs and DM regions DMRs (genomic clusters of ≥ 3 DM-CpGs and ≤ 1000 base pairs) were identified. Genes containing DMRs within their genomic boundaries were classified as DM genes. Positive and negative methylation differences coefficients refer to hypermethylation and hypomethylation, respectively.

### Bioinformatics and statistical analysis

**Figure 1:**
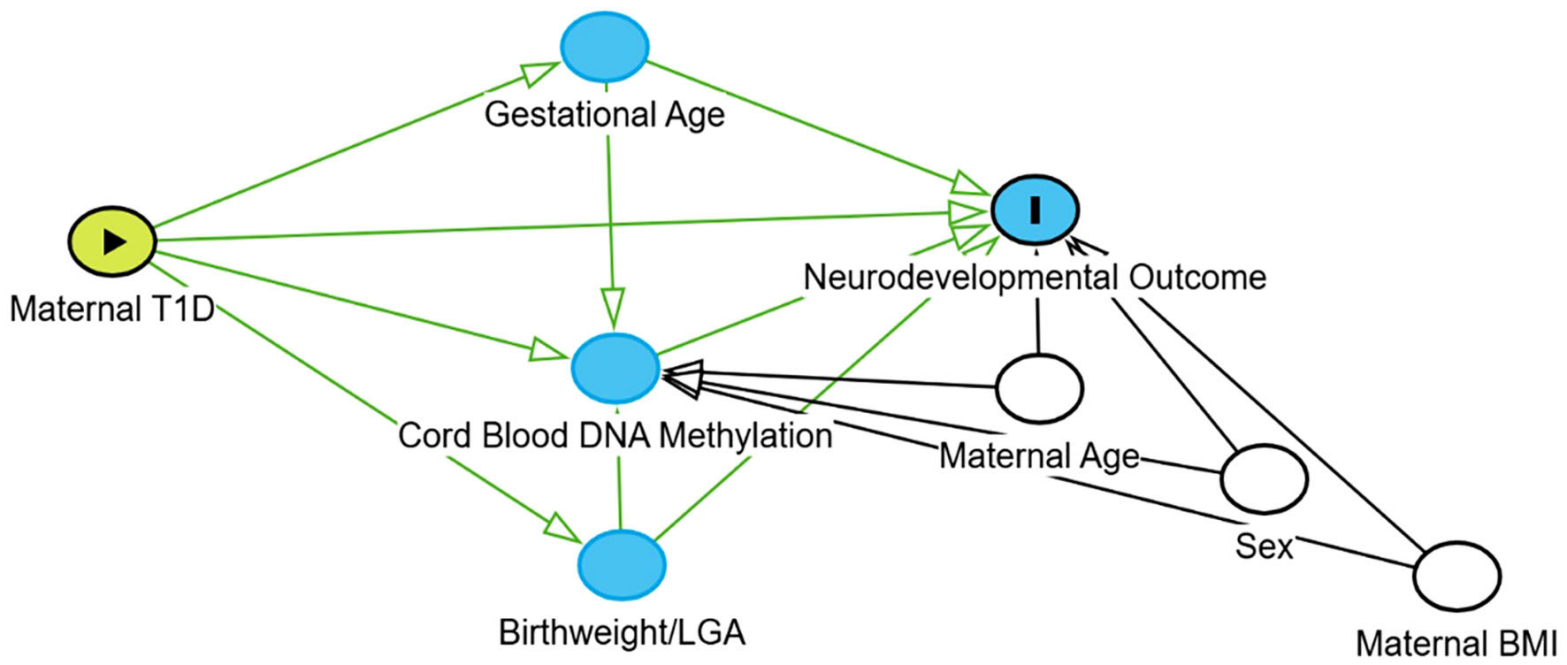
Directed acyclic graph (DAG) illustrating the hypothesized causal framework. This diagram outlines the assumed causal pathways connecting maternal type-1 diabetes to infant neurodevelopmental outcomes, mediated by intrauterine epigenetic modifications. Nodes represent variables, and arrows indicate hypothesized directional causal effects based on biological plausibility and literature.

Methylation analysis was conducted using the MethylKit R package (12). After loading the methylation data for each individual, a filter based on read coverage was applied to reduce bias associated with enrichment. Bases with coverage below 10X s and above the 99.9th percentile of coverage in each sample were discarded. For subsequent analyses, all samples were combined into a single object for base pair locations covered in all samples. The calculateDiffMeth function was used to identify CpG sites or regions with methylation differences using logistic regression. To clarify the hypothesized causal structure and justify the selection of covariates, a Directed Acyclic Graph (DAG) was developed (**Figure 1**). Based on this framework, maternal age, infant sex, and pre-pregnancy BMI were treated as confounders and included in the models. Although matching was performed using BMI categories, pre-pregnancy BMI was treated as a continuous variable in the regression models to account for residual intra-category variance and to provide a more precise adjustment for this potential confounder. Gestational age was included as a covariate to adjust for tissue maturity and isolate the direct effect of maternal type-1 diabetes on the methylome. Conversely, birthweight and LGA status were identified as downstream mediators and were excluded from the primary models to avoid overadjustment and collider bias.

P-values were adjusted to q-values to control the false discovery rate (FDR) in multiple hypothesis testing using the Sliding Linear Model (SLIM). The MethSeg function was applied to create CpG segments with similar profiles of differential methylation. Genomation R package and data obtained from the Hg19 known Genea BED-file genome browser website (13), was used for gene location and promoters, introns, exons, and intergenic regions identification.

CpGs sites were considered differentially methylated (DM) if differences were > 15% and the q-value < 0.01. Promoters, regions and islands were considered DM if differences were ≥ 1% and q-value < 0.01. DM CpGs and DM regions DMRs (genomic clusters of ≥ 3 DM-CpGs and ≤ 1000 base pairs) were identified. Genes containing DMRs within their genomic boundaries were classified as DM genes. Positive and negative methylation differences coefficients refer to hypermethylation and hypomethylation, respectively.

### Gene-set enrichment analysis

The Gene Ontology (GO) Platform (14) was used to conduct trait enrichment analysis on the DM-genes to identify association with neurodevelopment and brain functions. Pathways with FDR-corrected q-value <0.05 were considered statistically significant.

The overlap between DM genes enriched for neurological functions and the SFARI (15) gene database, which is the largest platform containing genes with mutations associated with ASD, was assessed. Furthermore, to facilitate the comparison between the top-100 SFARI genes and all DM genes identified in the IMT1-diabetes, a table comprising the names and coordinates of all human genome genes (Hg19, University of Santa Cruz, California) (13) was generated.

### Neurodevelopment assessment

Children were examined using the Bayley Scales of Infant Development-Third Edition (Bayley-III) (16) at two years of age (range 24–26 months) and the screening for ASD was performed using the M‐CHAT‐R-F test (17). Neurodevelopmental data were collected and analyzed by two psychometricians blind to whether the children were IMT1-diabetes or IHM. Partial correlation analysis was used to assess the relationship between the methylation values of DMRs and the different neurodevelopmental domains included in the Bayley test. To account for the influence of clinical group and gestational maturity on both DNA methylation and neurodevelopment, all correlation analyses between methylation values of DMRs and Bayley-III scores were performed using partial correlations adjusted for group (control and Type I Diabetes) and gestational age.

## Results

Maternal characteristics and perinatal outcomes are shown in **Table 1**. The mean glycated hemoglobin values were 6.9% ± 1.2 SDs; 6.5% ± 1.3 SDs and 6.6% ± 1.0 SDs, in the first, second and third trimester, respectively. Non obstetric or perinatal complications were recorded.

**Table 1:** Maternal characteristics and perinatal outcomes in the group of infants of mothers with type-1 diabetes (IMT1-diabetes) and infants of healthy mothers (IHM).

Variable	IMT1-diabetes (n = 10)	IHM (n = 10)	p-value	Total (n = 20)
Maternal characteristics				
Age (years)	32.3 ± 5.8	28.6 ± 4.8	0.138	30.5 ± 5.5
Pre-pregnancy weight (kg)	67.6 ± 12.8	62.6 ± 9.3	0.334	65.1 ± 11.2
Weight increase (kg)	12.4 ± 3.3	15.0 ± 4.2	0.133	13.7 ± 3.9
Height (cm)	164.9 ± 6.4	161.2 ± 6.3	0.209	163.1 ± 6.5
Body mass index (kg/m^2^)	24.7 ± 3.4	24.2 ± 3.7	0.721	24.4 ± 3.5
Body mass index, n (%)			0.470	
Normal weight	4 (40)	5 (50)		9 (45)
Overweight	6 (60)	4 (40)		10 (50)
Obesity	0 (0)	1 (10)		1 (5)
Race/Ethnicity (n, %)				
White/Caucasian	10 (100)	10 (100)	1.000	20 (100)
Parity = 0, n (%)	6 (60)	8 (89)	0.628	14 (70)
Perinatal outcomes				
Cesarean, n (%)	5 (50)	5 (50)	1.000	10 (50%)
Gestational age (days)	264.7 ± 4.7	276.2 ± 4.2	<0.001	270.5 ± 7.3
Newborn weight (g)	3574 ± 480	3262 ± 203	0.082	3418 ± 393
Weight percentile (%)	82.2 ± 24.0	57.2 ± 18.8	0.019	69.7 ± 24.6
LGA n (%)	6 (60)	0 (0)	0.011	6 (30)
Sex = Female, n (%)	6 (60)	8 (80)	0.628	14 (70)
Apgar test at 1^st^ minute	8.3 ± 1.6	9.0 ± 0.0	0.209	8.7 ± 1.2
Apgar test at 5^th^ minute	8.8 ± 0.6	9.1 ± 0.3	0.196	9.0 ± 0.5

Results are shown as percentages or mean and standard deviation. LGA, large for gestational age.

Data normality was assessed by inspecting the frequency distributions and the Kolmogorov–Smirnov test. Differences were studied using Student’s t-test. Comparison between proportions was performed using the chi-squared test and the Fisher’s exact test with small sample sizes (< 5). Statistical analyses were performed with SPSS v.21.0 (Chicago, IL).

### Genome-wide methylation analysis

**Figure 2:**
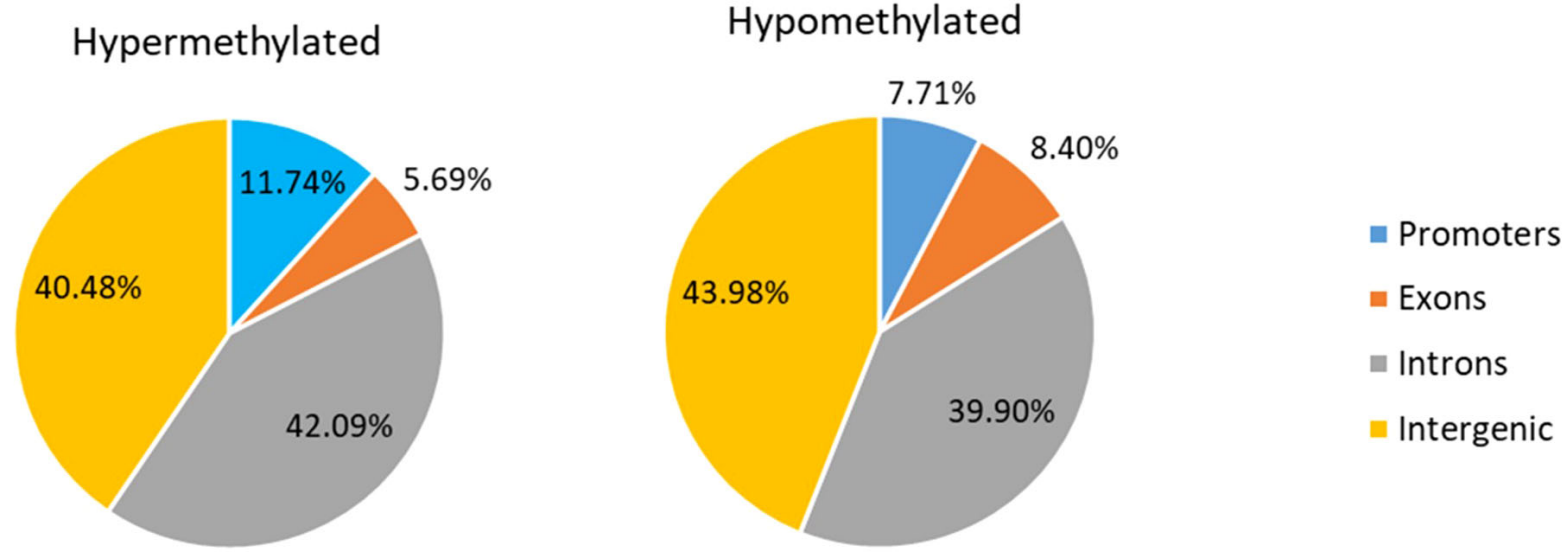
Differences in DNA methylation in promoters, intronics, exonics, and intergenic regions of IMT1-diabetes (hypo and hypermetylation, + or - methylation differences > 1% and q-value < 0.01).

From the 2,259,717 individual CpGs analyzed in each newborn, 16,018 resulted DM (methylation difference >15%, q < 0.01). Of all DM CpGs, 57.9% were hypomethylated, of which 11.74% were located in promoters, 5.69% in exons, 40.48% in introns and 42.09% in intergenic regions. Conversely, the remaining 42.1% CpGs, were hypermethylated, with 7.71% located in promoters, 8.40% in exons, 43.98% in introns and 39.90% in intergenic regions. In total we identified 7,150 DM promoters, 23,928 islands, and 3,647 regions DMRs (methylation difference > 1%, q < 0.01). **Figure 2**. The DMRs were annotated to a total of 1,127 unique genes.

### Gene-set enrichment analysis

Functional analysis was performed on the 1,127 genes containing DMRs, of which 920 were unequivocally mapped to the human genome. These genes were linked to 110 principal pathways (Supplementary material, **Supplementary Table S1**), with a significant association to critical neurodevelopmental pathways: “Vocal, Imitative, and Observational learning”, “Synapse organization” and “Neurogenesis”. **Table 2**. The genes involved in these three pathways are shown in **Table 3**. *NRXN1* and *SHANK3* were enriched across all neurodevelopmental pathways. *NRXN2* and *KIRREL3* were involved in two pathways each. Notably, the largest methylation differences were concentrated in three key genes: MYT1L (21.61%, q = 1.97E-07), NRXN1 (12.305%, q = 5.04E-75), and SHANK3 (11.620%, q = 9.81E-06). (Supplementary material, **Supplementary Table S2** provides specific details on these genes, including their locations, methylation differences and q values.

**Table 2:** Neurodevelopmental pathways associated with differentially methylated genes (n=1,127) in infants exposed to maternal Type 1 diabetes.

Biological processes	Homo sapiens, n Refs.	Genes n	Expected	Fold enrichment	+/-	q-value	FDR
Vocal learning	6	4	0.28	14.50	+	6.18E-05	1.27E-02
Imitative learning	6	4	0.28	14.50	+	6.18E05	1.29E-02
Observational learning	7	4	.32	12.43	+	1.39E-04	2.32E-02
Synapse organization	368	36	16.92	2.13	+	2.23E-05	6.69E-03
Neuron projection guidance-Neurogenesis	1380	94	63.43	1.48	+	1.38E	2.33E-02

Refs: number of references, FDR, False discovery rate (FDR) p < 0.05, methylation difference > 0.1%, q-value < 0.01.

**Table 3:** Genes with annotated DMRs involved in neurodevelopmental pathways.

Vocal imitative and observational learning DM genes n = 4	Synapse organization DM genes n = 38	Neuron projection guidance-neurogenesis DM genes n = 94		
NRXN1 ***	*ABI3*	ABI3**	*EPHA2*	***PTK7***
NRXN2 **	ADGRL3**	ADGRL3**	*EPHA8*	*PTPRG*
SHANK3 ***	*ACTL9*	*ADGRG1*	*FARP2*	*PTPRJ*
*STRA6*	*ADD2*	*AKT1*	*FGF1*	***PTPRT***
	*ACTN1*	***ARHGEF10***	*FGF12*	***ROBO2***
	*ARHGAP39*	*AZU1*	*FIG4*	*ROR2*
	*CACNA2D2*	*B4GALT5*	*FRY*	*RUNX3*
	***CACNG2***	*BAIAP2*	***FRYL***	*SCRIB*
	CDH1**	***BCL11B***	FZD1**	*SEMA3A*
	CNTNAP1**	*BCL2*	*FZD10*	*SEMA4D*
	***CX3CR1***	*BMP4*	*FZD7*	*SEMA5B*
	CYFIP1**	*BSG*	GABRA5*	*SEMA6B*
	DOCK10**	*CD9*	*GLI2*	*SEMA6C*
	FZD1**	CDH1**	*GPC2*	*SH3TC2*
	GABRA5**	*CDH4*	KIRREL3**	SHANK3***
	*GABRB3*	*CELSR1*	*LAMA2*	SLC1A1**
	*IGSF21*	CNTNAP1**	MDGA1**	***SOX5***
	*INSR*	*CRB2*	*MESP1*	*SPOCK1*
	*ITGAM*	*CSNK1D*	***MYT1L***	*SUN1*
	***ITGB3***	***CYFIP1***	*NECTIN1*	*TBCD*
	KIRREL3**	*DAB2IP*	***NOTCH1***	*TMEM132E*
	*MDGA1*	***DAGLA***	***NR4A2***	*TOR1A*
	***NLGN2***	*DAGLB*	NRXN1***	*TSPAN2*
	NRXN1***	***DLL1***	*OGDH*	*TUNAR*
	NRXN2**	DOCK10**	*PACSIN1*	*USH1C*
	*PALM*	*DSCAML1*	*PFKFB3*	*USH1G*
	*PLXNC1*	*EFHD1*	*PHGDH*	*VCL*
	*PLXND1*	*EFNA1*	*PLPP3*	WNT7B**
	***PTK7***	*EFNB1*	PLXND1**	*ZEB2*
	SHANK3 ***	*EFNB2*	*PRKCZ*	***ZMIZ1***
	***SLC1A1***	*EPHA10*	*PTCH1*	
	*SNTA1*		*PTK2*	
	*SPTBN2*			
	*WNT7B*			

Highlighted in red, differentially methylated genes involved in two and three pathways, ** and ***, respectively. *KIRREL3* and *MYT1L* exhibited the largest methylation differences. Gene names in bold indicate genes that overlap with those previously associated with autism spectrum disorders (ASD), according to the SFARI database (15).

Twenty of the genes enriched for neurodevelopment overlapped with ASD-linked genes from the SFARI database (15). **Table 3**. Moreover, other eight genes with annotated DMRs were identified as strong candidates for ASD according to the SFARI database: *ANKRD11, CACNA1C, CHD7, EHMT1, EBF3, FOXP1, KDM6B*, and *KCNQ2*.

### Neurological assessment of infants

**Figure 3:**
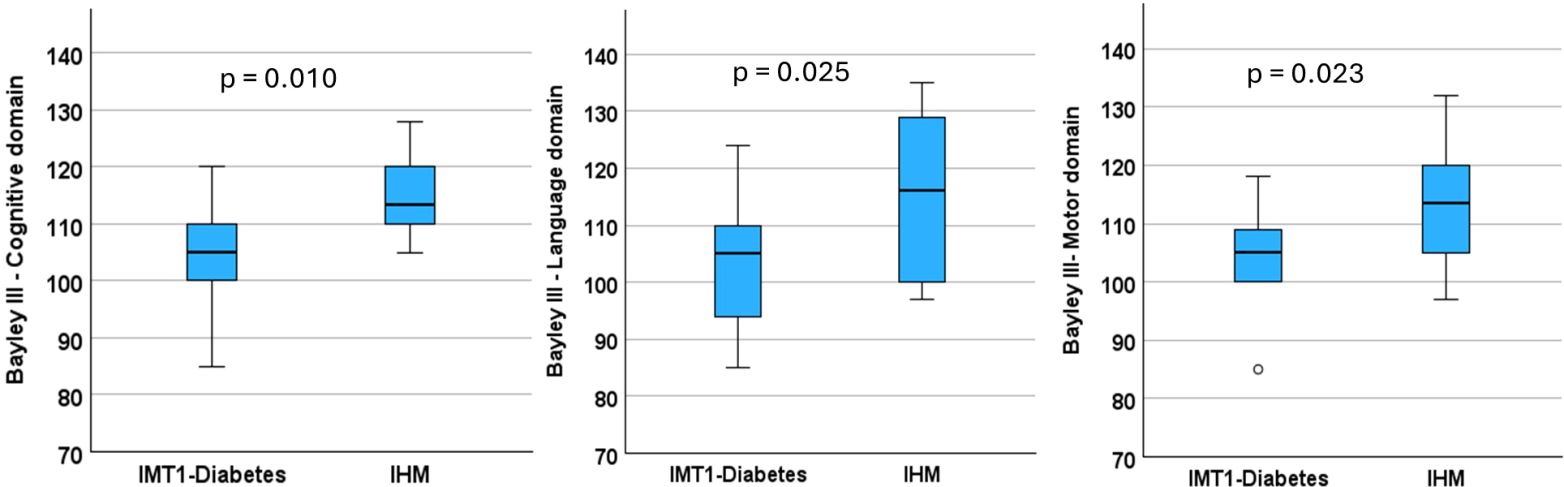
Boxplots showing cognitive, language, and motor domain scores on the Bayley-III-scales for infants exposed to maternal type 1 Diabetes (IMT1-diabetes) and infants born to healthy mothers (IHM).

At two years of age, IMT1-diabetes showed significantly lower scores than controls in cognitive (p = 0.010), language (p = 0.025), and motor (p = 0.023) domains on the Bayley-III scales. **Figure 3**. The screening test for ASD performed at this age was negative in all infants.

**Figure 4:**
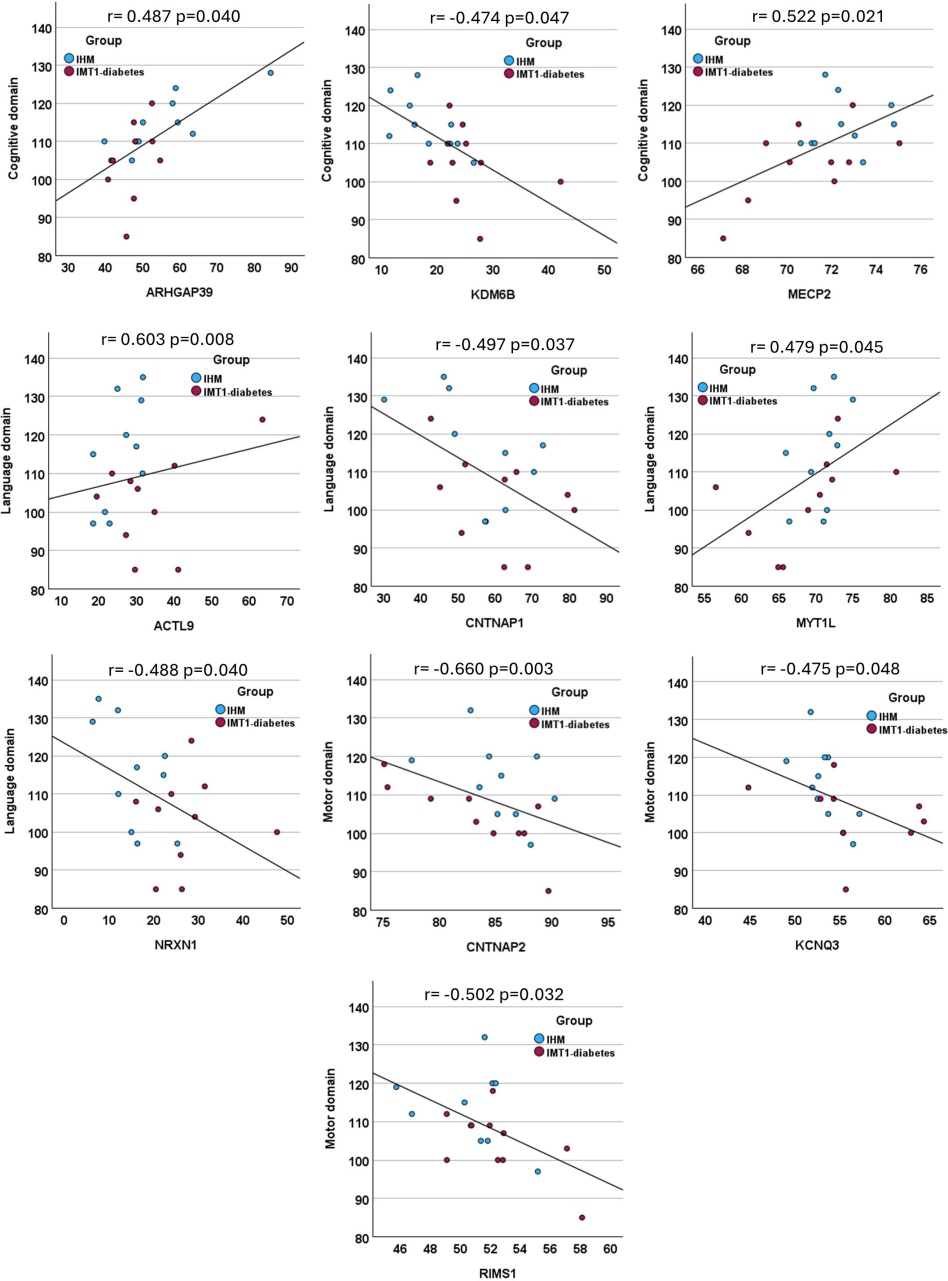
Correlations between Bayley scales of infant neurodevelopment and methylation values (%) of regions within differentially methylated genes. P-values and correlation coefficients (r) were derived from partial correlation analyses adjusted for group and gestational age.

**Correlations:** Partial correlation analysis revealed significant associations between Bayley-III scores and methylation values across the DM CpGs DMRs annotated to 10 genes. Cognitive domain scores correlated with methylation values in loci included in *ARHGAP39, KDM6B, and MECP2.* Language domain scores correlated with methylation values in loci contained within ACTL9, *CNTNAP1, MYT1L* and *NRXN1*. Moreover, motor domain scores correlated with methylation values in loci included in *CNTNAP2, K*CNQ3, and *RIMS1.* **Table 4** and **Figure 4**.

**Table 4:** Partial Correlations between Bayley-III neurodevelopmental scores and methylation values at differentially methylated loci adjusted for group and gestational age.

Gene	Genomic annotation	Domain r and (p-value)		
		Cognitive	Language	Motor
*ACTL9_r*	chr19:8808334-8808829	-0.362 (0.140)	0.603 (0.008)	0.406 (0.094)
*ARHGAP39_r*	chr8:145755105-145756129	0.487 (0.040)	-0.152 (0.548)	0.040 (0.875)
*CNTNAP1_r*	chr17:40836179-40837073	-0.182 (0.470)	-0.497 (0.037)	-0.239 (0.340)
*CNTNAP2_i*	chr7:147005399-147244794	-0.153 (0.543)	-0.142 (0.575)	-0.660 (0.003)
*KCNQ3*	chr8:133133109-133493342	-0.042 (0.867)	0.146 (0.562)	-0.475 (0.048)
*KDM6B_r*	chr17:7754919-7755044	-0.474 (0.047)	0.358 (0.144)	0.333 (0.177)
*MECP2*	chrX:153287025-153363174	0.522 (0.021)	0.266 (0.286)	-0.168 (0.505)
*MYT1L_r*	chr2:1870141-1878396	-0.369 (0.132)	0.479 (0.045)	-0.177 (0.483)
*NRXN1_r*	chr2:50201283-50201571	-0.223 (0.373)	-0.488 (0.040)	-0.018 (0.945)
*RIMS1*	chr6:72596254-73112847	-0.008 (0.976)	-0.114 (0.653)	-0.502 (0.032)

Subscripts appended to gene names (_r or _i) indicate whether the differentially methylated region values correlating with neurodevelopmental domains overlap with regions or islands, respectively. Highlighted in red, p<0.05.

## Discussion

This hypothesis-generating pilot study provides the first evidence of an association between neurodevelopmental alterations in offspring of mothers with Type 1 Diabetes and epigenetic changes detected in umbilical cord blood at birth. Children in the IMT1-Diabetes group scored significantly lower across the cognitive, language, and motor domains of the Bayley-III Scales. Importantly, we found that methylation values within DMRs annotated to ten crucial genes for neurodevelopment and brain function correlated significantly with these domain scores. The enrichment analysis confirmed that DMR-containing genes were significantly overrepresented in key neurological pathways, such as, Synaptogenesis, Neurogenesis Vocal, and Observational learning and Imitative learning. The largest methylation differences were concentrated in the neurodevelopmental genes *MYT1L*, *NRXN1*, and *SHANK3*. Both, *NRXN1* and *SHANK3*, were enriched across all identified neurodevelopmental pathways, and, notably, *NRXN1* and *MYT1L* showed significant correlation with the Bayley-III language domain score.

Arabiat et al. (18) in 2024 utilized the Bayley Scales to demonstrate an association between maternal Diabetes and poorer mental and motor skill outcomes in children aged 12–24 months. We have previously observed abnormal neurodevelopment features in video-EEG recording of infants of mothers with GDM and Type 1 Diabetes (19), difficulties in establishing stable behavioral states (20) and lower memory and learning abilities, both *in utero* and after birth (21). Levels of glycosylated hemoglobin were associated with the observed neurodevelopmental alterations (19–21). In the present study, birth weight, and the rate of large-for-gestational-age newborns, were significantly higher in the IMT1-diabetes group, objectively confirming the provided effect of maternal hyperglycemia on intrauterine growth (22).

To date, the few existing studies examining the link between intrauterine DNA methylation changes and subsequent neurodevelopmental outcomes in children have focused exclusively on GDM, not Type 1 Diabetes. Recently (2025), Wang et al. (23) demonstrated in rodent models that high glucose exposure alters DNA methylation and gene expression in neural progenitor cells. These alterations affected pathways critical for neurogenesis, axon guidance, and synaptic development, which provides a plausible biological basis for the observed neurodevelopmental impairment and aligns with the findings of our study.

Severe overlaps were observed between genes with DMRs that we identified in cord blood samples of IMT1-Diabetes and those reported in prior epigenetic studies of placental samples of pregnant women with GDM: *NRXN1* and *PRDM16* overlapped with genes reported Wang et al. (24), but they did not find any enriched pathway accounting for 256 differentially methylated genes that identified. *ABLIM1* and *SASH1* overlapped with genes identified by Chen et al. (25). In addition, *SHANK3* and *GRIK4* were differentially methylated in both our cord blood cohort and the placental cohort studied by Lu et al. (26). None of these prior studies assessed the correlation between the identified methylation changes and the subsequent neurodevelopment of the children. Also, in placental samples (but not those from pregnancies complicated by diabetes), Diez-Ahijado et al. (27) in 2024 are the sole investigators to have reported an association between methylation levels in DMRs annotated to four neurological development genes and cognitive development in children. One of these, *MECOM*, was also identified in our analysis. However, the enrichment analysis of all DM genes they identified did not reveal associations with neurological development pathways, a finding that contrast with the clear demonstration achieved in our study.

Our analysis identified 28 genes with annotated DMRs in the IMT1-diabetes group that overlapped with genes associated with ASD, according with the SFARI database (15). A large 2024 epigenome-wide association meta-analysis by Schuurmans et al. (28) identified DNA methylation differences associated with ASD using polygenic scores on cord blood DNA, but reported no overlap of differentially methylated candidate genes for ASD, either within their cohorts or with other published studies. To date, no studies have linked epigenetic changes in cord blood DNA with a later ASD diagnosis in children exposed to maternal Type-1 Diabetes. Only Howe et al. (29) previously explored this possibility in children exposed to GDM, identifying methylation differences in *OR2L13*, a neurodevelopment-associated gene. Notably, *OR2L1*3 was also identified in our analysis. Furthermore, five neurodevelopment-enriched genes from our analysis (*CNTNAP2, GALNT, SLC4A10, KIRREL3*, and *MYT1L*) coincided with the DM genes identified by Mordaunt et al. (30) in 2025 in the cord blood of females later diagnosed with ASD, all of which are included the SFARI database (15). In our study, *KIRREL3* and *MYT1L* exhibited the largest methylation differences; and *MYT1L* correlated with the Bayley-III language domain score, and *CNTNAP2* correlated with the motor domain score. As ASD is a neurodevelopmental disorder, the involvement of genes associated with brain development is expected.

Among the strengths of this work is it originality as the first prospective, hypothesis-generating pilot study to suggest that epigenetic changes identified in newborns exposed to maternal Type1 Diabetes are potentially linked to later neurodevelopmental outcomes in childhood. Furthermore, we used WGBS, the gold-standard technique for DNA methylation analysis, which covered over 3.34 million CpGs. This method provides a more complete and unbiased view of the methylome compared to array-based studies (such as the Illumina 450-BeadChip or EPIC array, which cover 400-752,000 CpG sites). The broader coverage explains why we identified a higher number of DM CpGs and DMRs than similar previous studies. The only exception was the Mordaunt et al. (30) study, with which we found the most overlap, likely because they also used a WGBS approach.

The primary limitation of this study is its small sample size, which may increase the risk of false-positive findings; consequently, the present results should be interpreted as exploratory. This work does not constitute a definitive epigenome-wide association study (EWAS), but rather serves to identify potential pathways for future research. Due to sample size constraints, several key factors—including cell-type heterogeneity, maternal glycemic control, socioeconomic status, and education level—were not incorporated into the regression models to avoid over-parameterization. Nevertheless, these findings provide a valuable foundation for future larger-scale research that should account for these variables alongside gene expression analysis. If confirmed, DNA methylation patterns in umbilical cord blood could serve as potential biomarkers for neurodevelopmental alterations, enabling targeted interventions for high-risk newborns.”

In conclusion, while further confirmation in much larger cohorts is needed, we provide the first results supporting the hypothesis that neurodevelopmental alterations observed in children born to mothers with Type 1 Diabetes are potentially linked to DNA methylation changes during intrauterine life, which can be identified at birth.

## Data Availability

The datasets presented in this study can be found in the article/**Supplementary Material**. Further inquiries can be directed to the corresponding author/s.
