# Supplementary material for: Infants exposed to maternal type 1 diabetes: intrauterine epigenetic modifications and neurological development
Source: Front Endocrinol (Lausanne). 2026 Feb 10;17:1759949. doi: 10.3389/fendo.2026.1759949 (PMC12929157; doi:10.3389/fendo.2026.1759949)

**Table S1.** Gene Ontology (GO) enrichment analysis of differentially methylated genes of infants exposed to maternal type 1 diabetes versus control infants^16^.


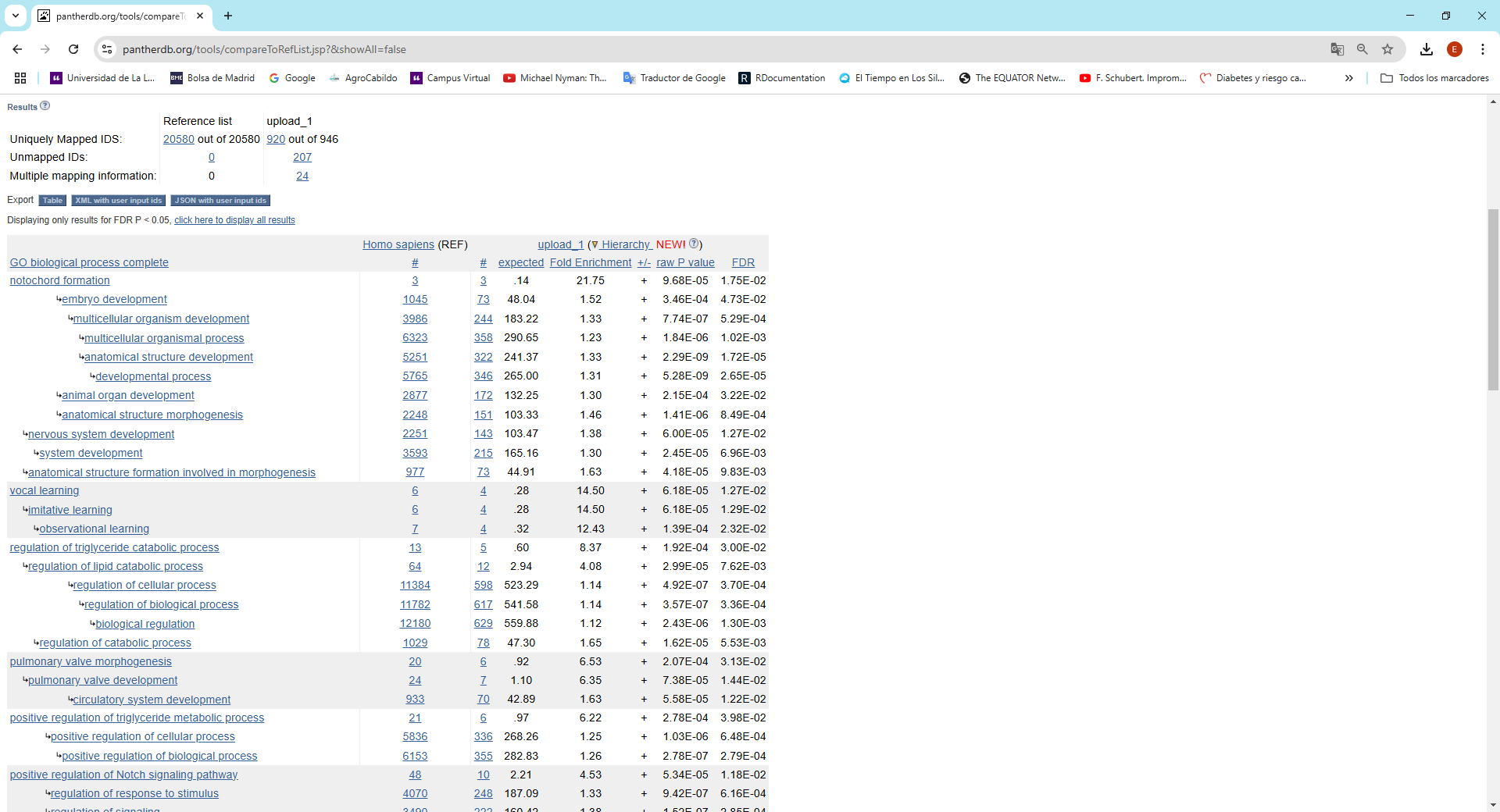

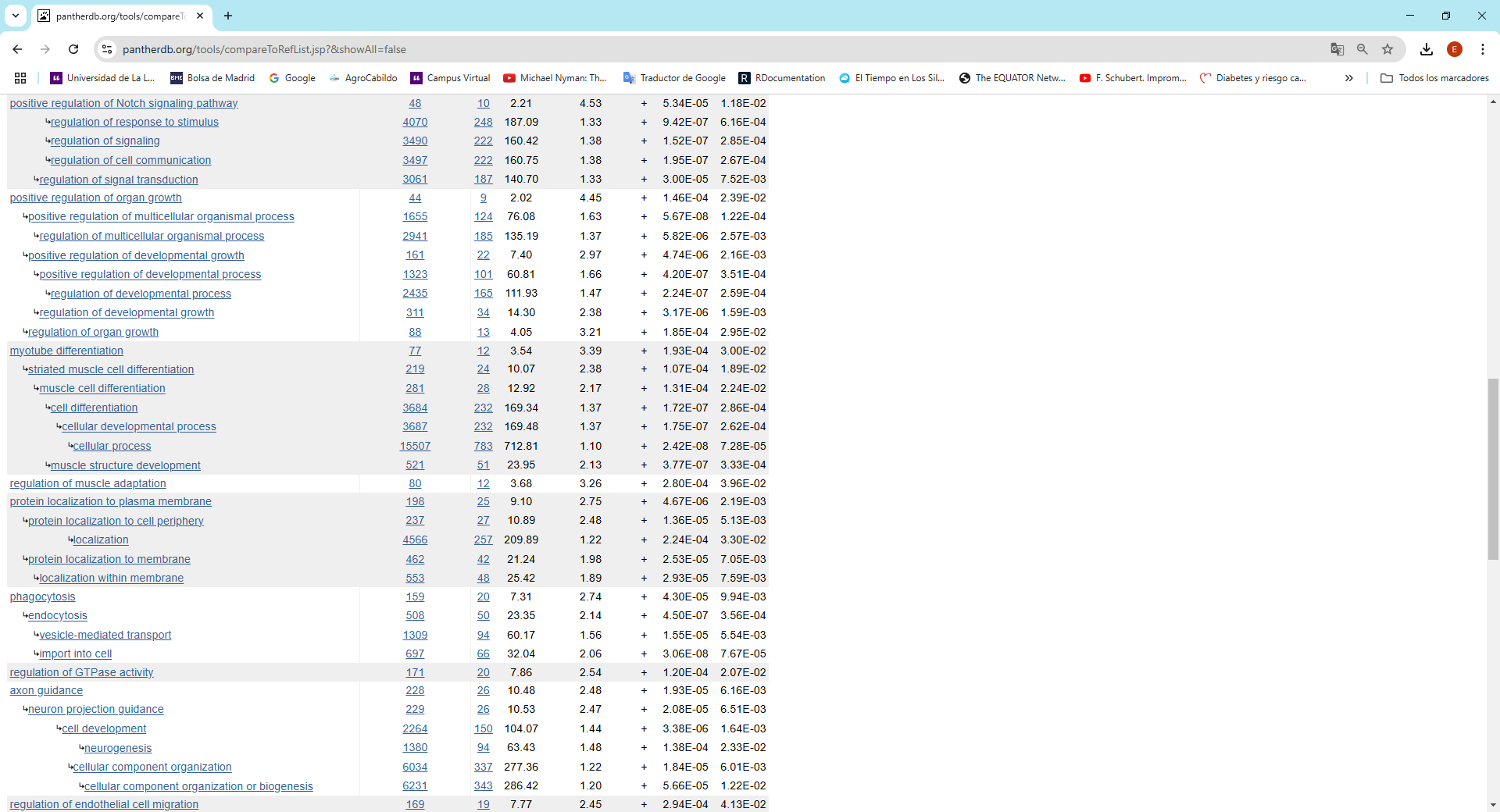

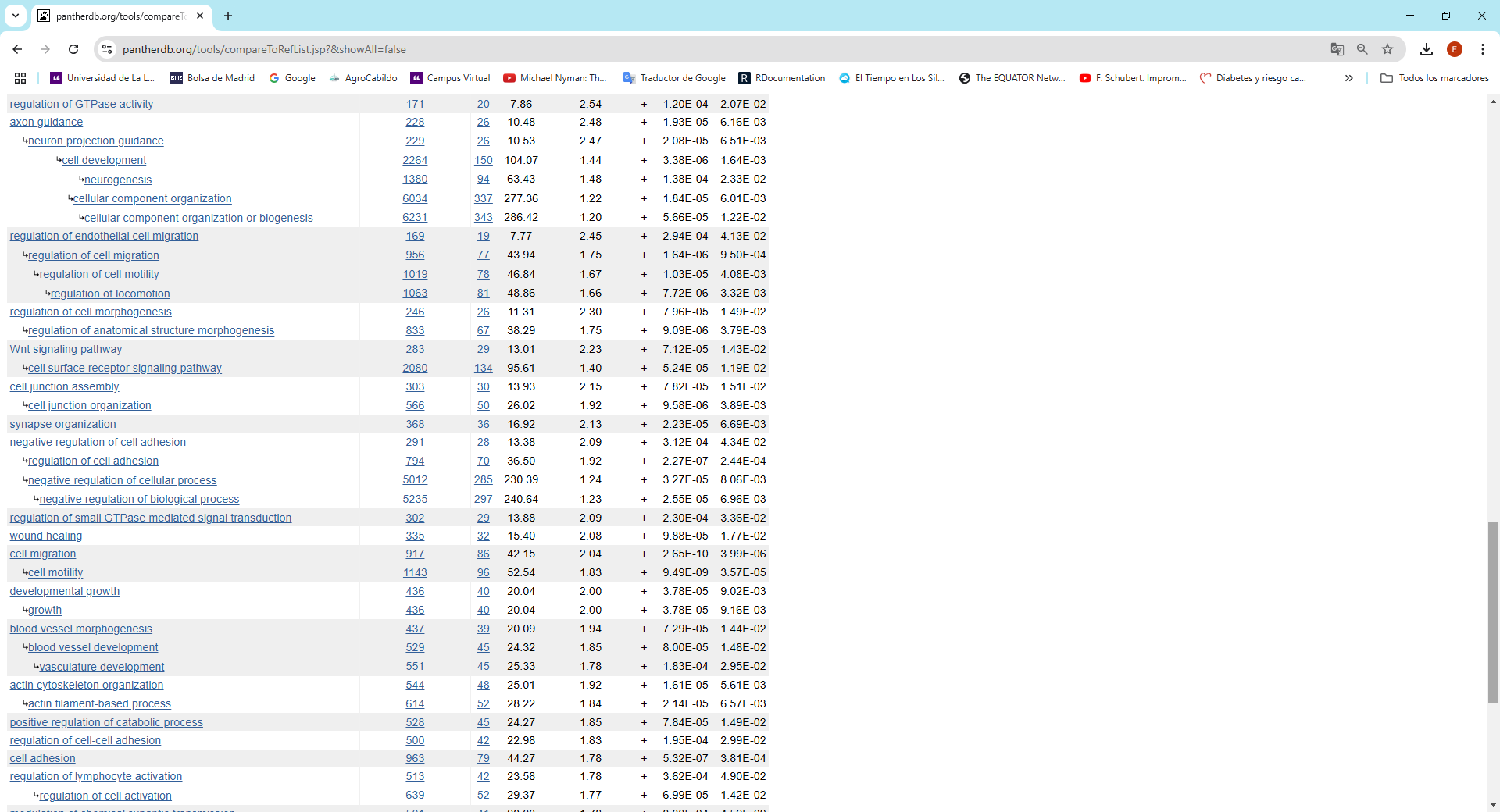

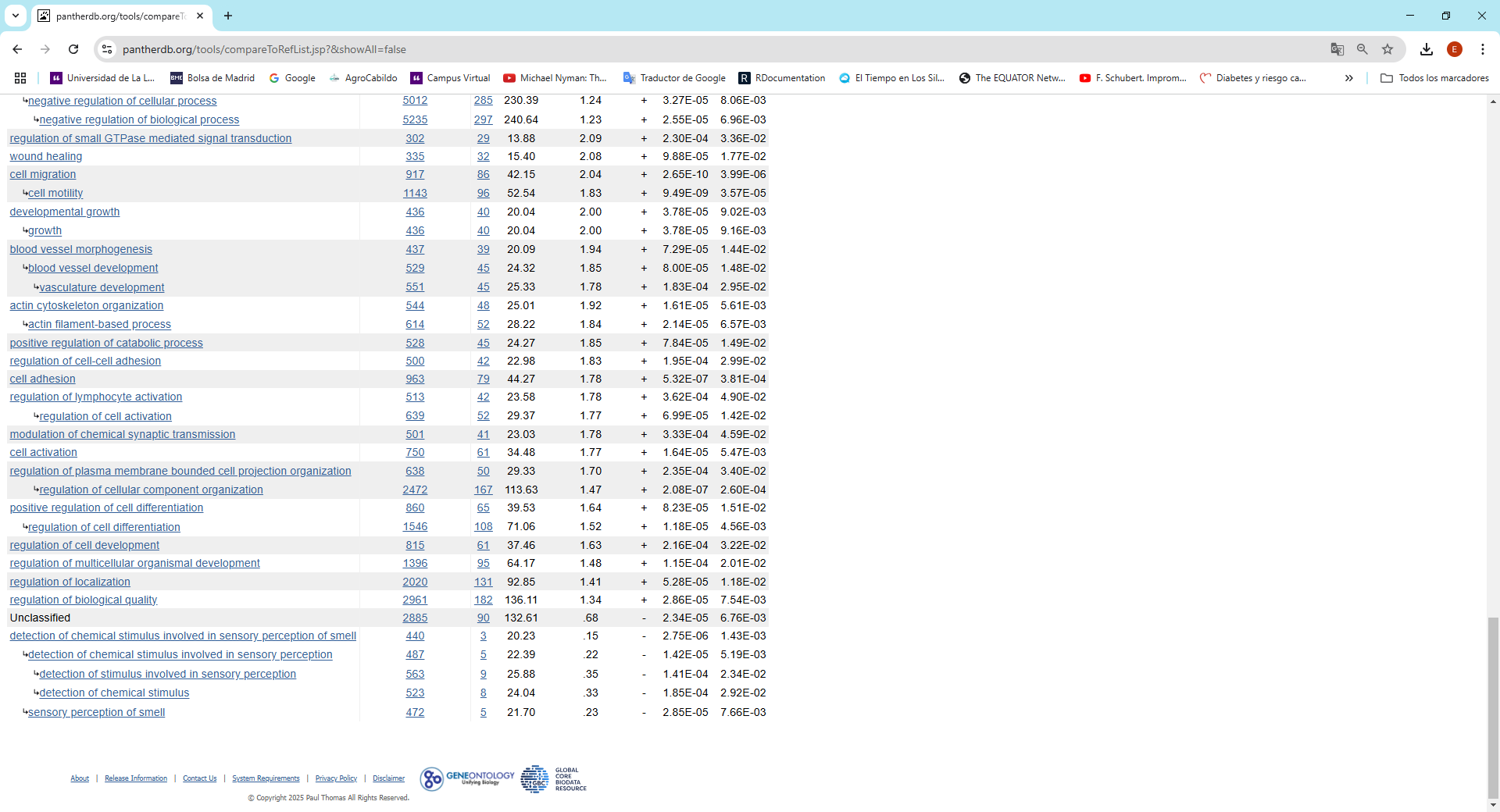

Supplement: Supplementary file 1 [file Table1.docx]
