# Supplementary material for: Infants exposed to maternal type 1 diabetes: intrauterine epigenetic modifications and neurological development
Source: Front Endocrinol (Lausanne). 2026 Feb 10;17:1759949. doi: 10.3389/fendo.2026.1759949 (PMC12929157; doi:10.3389/fendo.2026.1759949)
Supplement: Supplementary file 2 [file Table2.docx]

Table S2. Genes with annotated differentially methylated regions (DMRs) involved in each neurodevelopmental pathways. DMRs location, methylation differences values, and q-values. In bold, genes associated with all pathways and/or with the greater MD, and/or multiple DMRs.

| **Vocal Imitative and Observational learning** | | | | | **Synapse organization** | | | | | **Neuron projection guidance-Neurogenesis** | | | | |
| --- | --- | --- | --- | --- | --- | --- | --- | --- | --- | --- | --- | --- | --- | --- |
| Gene | Gene location | DMR location | MD (%) | q-value | Gene | Gene location | DMR location | MD (%) | q-value | Gene | Gene location | DMR location | MD (%) | q-value |
| **NRXN1** | chr2:50145640-51259270 | chr2:50201283-50201571 | **12.305** | **5.04E-75** | **NRXN1** | **chr2:50145640-51259270** | **chr2:50201283-50201571** | **12.305** | **5.04E-75** | **NRXN1** | chr2:50145640-51259270 | chr2:50201283-50201571 | **12.305** | **5.04E-75** |
| **SHANK3** | chr22:51111250-51171640 | chr22:51142803-51142935 | **11.620** | **9.81E-06** | **SHANK3** | **chr22:51111250-51171640** | **chr22:51142803-51142935** | **11.620** | **9.81E-06** | **SHANK3** | chr22:51111250-51171640 | chr22:51142803-51142935 | **11.620** | **9.81E-06** |
| **NRXN2** | chr11:64373645-64490669 | chr11:64400792-64406046 | **-5.640** | **1.62E-10** | **NRXN2** | **chr11:64373645-64490669** | **chr11:64400792-64406046** | **-5.640** | **1.62E-10** | **MYT1L** | chr2:1792884-2330083 | chr2:1801585-1802171 | **21.061** | **1.97E-07** |
|  |  | chr11:64406051-64435019 | **-1.290** | **1.65E-09** |  |  | **chr11:64406051-64435019** | **-1.290** | **1.65E-09** | SOX5 | chr12:23682437-24715425 | chr12:23709904-23737433 | -6.240 | 4.28E-10 |
|  |  | chr11:64435066-64444973 | **-8.065** | **1.78E-20** |  |  | **chr11:64435066-64444973** | **-8.065** | **1.78E-20** | NR4A2 | chr2:157180948-157189233 | chr2:157181359-157184390 | -4.751 | 2.89E-08 |
|  |  |  |  |  | NLGN2 | chr17:7311163-7323179 | chr17:7311282-7311772 | 10.118 | 3.62E-05 | ARHGEF10 | chr8:1772091-1906807 | chr8:1850925-1861131 | -5.180 | 5.70E-05 |
|  |  |  |  |  | CACNG2 | chr22:36956903-37099797 | chr22:36960499-36961020 | 6.760 | 5.07E-08 |  |  | chr8:1870661-1870867 | 7.362 | 3.48E-52 |
|  |  |  |  |  | CX3CR1 | chr3:39304984-39323226 | chr3:39309390-39314574 | -9.233 | 4.80E-03 | BCL11B | chr14:99635624-99738534 | chr14:99679658-99681841 | 4.558 | 7.04E-03 |
|  |  |  |  |  | CYFIP1 | chr15:22892169-23006015 | chr15:22904503-22916009 | 5.959 | 6.10E-09 | CYFIP1 | chr15:22892169-23006015 | chr15:22904503-22916009 | 5.959 | 6.10E-09 |
|  |  |  |  |  | GABRB3 | chr15:26788693-27018223 | chr15:26915257-26915777 | 9.808 | 1.35E-28 | DAGLA | chr11:61447862-61514473 | chr11:61488268-61498848 | -5.719 | 1.21E-32 |
|  |  |  |  |  | ITGB3 | chr17:45331192-45391109 | chr17:45343767-45356509 | -5.363 | 6.89E-03 | DLL1 | chr6:170591293-170600166 | chr6:170597326-170597498 | 11.644 | 2.08E-05 |
|  |  |  |  |  | **KIRREL3** | chr11:126293252-126870666 | **chr11:126316942-126329507** | -6.166 | 1.14E-10 | FRYL | chr4:48499373-48782296 | chr4:48683251-48713145 | -5.603 | 5.65E-10 |
|  |  |  |  |  |  |  | **chr11:126634190-126787721** | **-7.353** | **1.53E-23** | **KIRREL3** | chr11:126293252-126870666 | chr11:126316942-126329507 | **-6.166** | **1.14E-10** |
|  |  |  |  |  | PTK7 | chr6:43044051-43129453 | chr6:43082296-43084276 | 10.488 | 3.15E-14 |  |  | chr11:126634190-126787721 | **-7.353** | **1.53E-23** |
|  |  |  |  |  | SLC1A1 | chr9:4490467-4587469 | chr9:4517892-4586301 | 7.898 | 1.32E-14 | NOTCH1 | chr9:139388884-139440500 | chr9:139405153-139406601 | -11.290 | 4.47E-45 |
|  |  |  |  |  |  |  |  |  |  | PTK7 | chr6:43044051-43129453 | chr6:43082296-43084276 | 10.488 | 3.15E-14 |
|  |  |  |  |  |  |  |  |  |  | PTPRT | chr20:40701395-41818546 | chr20:41249229-41307755 | -10.675 | 4.25E-05 |
|  |  |  |  |  |  |  |  |  |  | ROBO2 | chr3:76360030-77699114 | chr3:76891362-77076842 | -6.337 | 5.53E-43 |
|  |  |  |  |  |  |  |  |  |  | SLC1A1 | chr9:4490467-4587469 | chr9:4517892-4586301 | 7.898 | 1.32E-14 |
|  |  |  |  |  |  |  |  |  |  | ZMIZ1 | chr10:80828722-81076275 | chr10:81033980-81034368 | 6.786 | 1.04E-21 |
